# Abstract of the Proceedings of the Buffalo Medical Association

**Published:** 1871-03

**Authors:** Wm. C. Phelps

**Affiliations:** Secretary; Buffalo


ART. III.—*Abstract of the Proceedings of the Buffalo Medical Association.*

BUFFALO, Tuesday Evening, March 7th, 1871.

The President. Dr. S. W. WETMORE, in the Chair.

The Secretary being absent, Dr. F. W. Abbott was appointed Secretary, *pro tem.*

Dr. White announced the death of J. Herman Bird, M. D., of Sioux City, Iowa, formerly a resident of this city.

Dr. White said that Dr. Bird was a gentleman of brilliant attainments, and had made a wide reputation by his writings on cholera, in 1849 and 1851; and that he was the discoverer of the "sulphur cure" for this disease, which has been in extensive use. He spoke, also, of some very interesting articles on the climate of the Northwest, which Dr. Bird had recently published. He then moved that Drs. Abbott and Rochester be appointed a committee to draft resolutions expressing the sentiments of the meeting in reference to this loss to the profession.

Dr. Rochester seconded the motion, and said that Dr. Bird's reputation was not confined to this country, for his writings concerning the influence of ozone in cholera epidemics had been pubashed in the medical journals of England, France and Germany, is well as in those of this country.

The following resolutions were adopted:

*Whereas*, It has pleased Divine Providence to remove by death our former associate, Dr. J. Herman Bird.

*Resolved*, That we extend to the family of the deceased our sincere and heartfelt sympathy in their affliction, and that while they mourn the loss of one naturally endeared to them, we must express the loss of our profession and the community in the death of one whose researches in the cause of science has made his name to be known even to those who live in foreign lands and speak other languages.

Frank W. Abbott, M. D.
S. W. Wetmore, M. D.
Thos. F. Rochester, M. D.,
Committee.

On motion of Dr. White, it was resolved that copies of these resolutions be presented to the family of the deceased, and furnished to the *Buffalo Medical & Surgical Journal*, and to the city papers for publication.

Dr. Rochester moved that Dr. Eastman, who is at present residing in California—and Dr. Herman P. Babcock, of Oakland, California—be appointed delegates of this Association to the Annual Session of the American Medical Association to be held in San Francisco, May 2d, 1871.

Dr. STRONG moved, as an amendment, that the President and Secretary be authorized to issue credentials to such members as wished to attend as delegates to the uumber of our representation. Dr. Rochester accepted this amendment, and the resolution as amended was adopted.

Under the order of prevailing diseases, Dr. ROCHESTER reported influenza, typhoid fever, and diarrhoea, as being most frequently met in practice.

Dr. MINER remarked that he believed it the general impression, that a large number of cases of typhoid fever were occurring in the city, but from his observation he was convinced that not one in ten called such were true cases of that disease. They are of a malarial character, generally taking the form of remittent fever, occurring as a result of the open winter which we have passed through. It is due to climatic causes not at present understood by the profession. Typhoid fever is also, perhaps, more common than usual at this season, but is by no means so alarmingly frequent as generally supposed.

Dr. WHITE said that there was a needless fear in the minds of the residents of the city in regard to the impure character of the water furnished by the City Water Works. While he was not an advocate for the quality of the water furnished at times, still he was of the opinion that, at no time, was it such an active cause of disease as is generally supposed. The worst cases of diarrhœa now generally present, which he has seen, have been in families who used filtered rain water.

Dr. Strong was of opinion that much of the disease which has prevailed here this winter, and also last winter, is not true typhoid fever. While in Washington had heard of it. By a stretch of nomenclature it might be called typho-malarial fever, but it lacked the symptoms and pathological changes which are present in typhoid fever. It was, he thought, in a great measure due to the surface water finding its way into the system by our food and drink. During an open winter, on every warm day, there is more or less of this, which hold in solution a large amount of decaying matter, and thus becomes a cause of disease.

Dr. MINER suggested that the cause of remittent fever is not known, and is not limited to any particular locality. It is generally supposed to be most common near marshes, and other low, wet places containing decaying vegetable matter, but he had seen it in all localities and under all circumstances.

Dr. WETMORE had seen it in nursing children, and was of opinion that it was a disease due to climatic causes. Had also seen a case recently, of cerebro-spinal meningitis occurring in a child twenty months of age. No spots were visible till after death. It was said to be quite common in the locality where it occurred.

WM. C. PHELPS, Secretary.

Adjourned.

